# Chromosomal Location Determines the Rate of Intrachromosomal Homologous Recombination in Salmonella

**DOI:** 10.1128/mBio.01151-21

**Published:** 2021-06-01

**Authors:** Eva Garmendia, Gerrit Brandis, Lionel Guy, Sha Cao, Diarmaid Hughes

**Affiliations:** a Department of Medical Biochemistry and Microbiology, Biomedical Center, Uppsala University, Uppsala, Sweden; University of Utah

**Keywords:** nucleoid-associated proteins, *Salmonella*, chromosome organization, homologous recombination

## Abstract

Homologous recombination is an important mechanism directly involved in the repair, organization, and evolution of prokaryotic and eukaryotic chromosomes. We developed a system, based on two genetic cassettes, that allows the measurement of recombinational repair rates between different locations on the chromosome. Using this system, we analyzed 81 different positional combinations throughout the chromosome to answer the question of how the position and orientation of sequences affect intrachromosomal homologous recombination. Our results show that recombination was possible between any two locations tested in this study and that recombinational repair rates varied by just above an order of magnitude. The observed differences in rate do not correlate with distance between the recombination cassettes or with distance from the origin of replication but could be explained if each location contributes individually to the recombination event. The relative levels of accessibility for recombination vary 5-fold between the various cassette locations, and we found that the nucleoid structure of the chromosome may be the major factor influencing the recombinational accessibility of each chromosomal site. Furthermore, we found that the orientation of the recombination cassettes had a significant impact on recombination. Recombinational repair rates for the cassettes inserted as direct repeats are, on average, 2.2-fold higher than those for the same sets inserted as inverted repeats. These results suggest that the bacterial chromosome is not homogenous with regard to homologous recombination, with regions that are more or less accessible, and that the orientation of genes affects recombination rates.

## INTRODUCTION

Homologous recombination is the process by which two sequences that share a sufficient length of sequence similarity undergo informational exchange. Homologous recombination has often been studied in the context of horizontal genetic transfer, where recombination between an acquired foreign DNA fragment and a homologous region on the host chromosome results in the integration of the transferred fragment (1, 2). However, the pathways by which such recombination occurs are thought to have evolved as chromosomal repair mechanisms to facilitate sister-chromosome exchange (3, 4). These pathway studies have identified RecA as the main control protein for initiating homologous recombination, with RecBCD and RecFOR guiding the process along alternative pathways (3, 5, 6).

Homologous recombination between similar DNA sequences within a chromosome can result in coevolution of sequences by gene homogenization, as has been shown for gene families like the *tuf* and *nif* genes (7, 8). Another potential outcome is chromosomal rearrangements, namely, duplications or deletions when recombination occurs between direct repeats or inversions as a result of recombination between inverted repeats (9–11). These varied and seemingly opposite effects of homologous recombination (rearrangements contribute to chromosome plasticity and result in variability within bacterial populations, whereas homogenization of sequences reduces variation and avoids divergence) show the big role that intrachromosomal homologous recombination can have in the evolution of bacterial populations.

Recombination within a chromosome can potentially happen between any two identical sequences, although previous research has shown the existence of structural regions that affect the physical proximity of different parts of the chromosome (12, 13). This structuration of the bacterial chromosome might result in some regions undergoing intrachromosomal recombination with each other at a higher rate than others, but it is still unclear how the position and orientation of identical sequences affect the rate of recombination between them.

In this work, we have constructed an experimental system composed of two genetic cassettes based on mutationally inactivated kanamycin resistance genes (*kan*), which can give rise to a kanamycin-resistant bacterium if homologous recombination leads to the repair of one of the *kan* genes. With this method, we were able to measure recombinational repair rates between pairs of identical sequences located at different positions in the chromosome and in different orientations. Recombinational repair of the *kan* genes was RecAB dependent but RecF independent, similar to what has been seen previously for this kind of recombinational repair between the separated *tufA* and *tufB* genes (7, 14). The recombinational repair rates for 81 different combinations of chromosomal locations varied by little more than an order of magnitude. The observed differences in repair rates associated with different locations were not explained by the distance separating the gene cassettes from each other or by the distance to the origin of replication. Instead, we found that individual relative accessibility for recombination at each position could explain the measured recombination rates and that the accessibility of each location is affected by the local nucleoid structure. These results suggest that each region of the chromosome influences the rate of recombination independently of the location of the homologous copy.

## RESULTS

### Construction of a cassette to measure intrachromosomal recombination rates.

**FIG 1:**
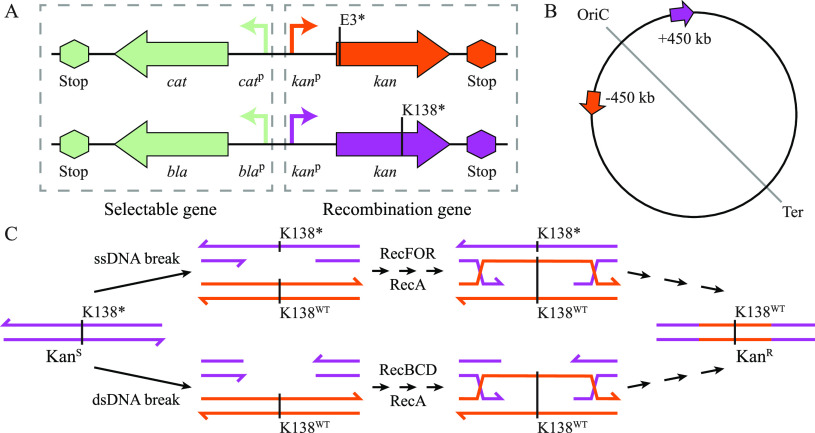
Overview of the experimental setup. (A) Stop codons were inserted into the beginning (orange) or the middle (purple) of a kanamycin resistance gene (*kan*). Each inactivated kanamycin resistance gene was paired with an active chloramphenicol (*cat*) or ampicillin (*bla*) resistance gene as a selectable marker for strain construction. *cat*^p^, *cat* promoter. (B) The recombination cassettes (orange and purple arrows) were inserted on the leading strand of replication on opposite sides of the replication origin, 450 kb from the origin of replication. (C) Basic model for the potential recombinational repair process. The process is initiated by a single-stranded DNA (ssDNA) or double-stranded DNA (dsDNA) break. The break is processed by RecFOR or RecBCD, followed by RecA-dependent strand invasion of the homologous *kan* gene. DNA synthesis followed by Holliday junction resolution and replication leads to the formation of kanamycin-resistant cells. The asterisk indicates a mutation to a nonsense codon--TAA in each case; K138^WT^, wild-type cassette sequence.

We constructed two genetic cassettes to measure rates of recombinational repair between identical sequences located at different positions on the bacterial chromosome. The cassettes are based on a pair of kanamycin resistance genes, each of which carries an inactivating stop codon at the beginning or the middle of the gene (Fig. 1A). Each *kan* gene is inactive in itself, but spontaneous homologous recombination between the two inactive *kan* genes can lead to an exchange of genetic information that can repair the sequence of either *kan* gene (Fig. 1C). The resulting cells produce an active aminoglycoside acetyltransferase protein and are kanamycin resistant. In addition to containing the inactive kanamycin resistance gene, each of the recombination cassettes contains an active ampicillin or chloramphenicol resistance gene as a selectable marker to facilitate selective insertion of the cassettes into various locations around the chromosome (Fig. 1A).

We initially inserted the two cassettes on the leading strand of replication, diametrically opposed on opposite sides of the replication origin, each 450 kb distant from *oriC* (Fig. 1B). This positioning of these cassettes is approximately the same as the positioning of the *tuf* genes (*tufA*, kb −480 from *oriC*; *tufB*, kb +280), for which recombinational repair has been studied extensively (7, 15, 16). To measure homologous recombination between these sequences, 100 independent cultures were grown overnight at 37°C in LB medium before aliquots containing approximately 2 × 10^5^ cells were spread on LA (LB with 1.5% agar; Oxoid) containing kanamycin to select putative recombinants that had repaired either kanamycin resistance gene (see Materials and Methods). This experiment is set up as a fluctuation assay, and the recombinational repair rate was calculated using the proportion of cultures in which no recombinants were scored (P_0_ method) (17). Successful recombination was observed in 59 cultures, which corresponds to a recombinational repair rate of 4.5 × 10^−6^ per cell per generation (Table 1). However, there are three potential sources of false positives that could generate kanamycin resistance in this selection, namely, (i) mutational reversion of either internal stop codon to the original sense codon, (ii) mutational change of either internal stop codon to an alternative codon that leads to the production of an active protein, and (iii) extragenic stop codon suppressor mutations. To measure the combined rate of all false positives, we constructed two strains that each carried only one of the respective recombination cassettes and measured the rate of spontaneous kanamycin resistance in the absence of a potential recombination partner gene. Each of the recombination cassettes displayed a spontaneous kanamycin resistance rate of about 2.4 × 10^−9^ (Table 1). This rate of false positives is 1,000-fold smaller than the measured rate of recombinational repair. It is therefore unlikely that false positives contribute significantly to the measured rate of recombinational repair. To further validate our results for the recombinational repair rate, we sequenced both *kan* genes in each of the 59 isolates that were selected during the measurement (see Table S1 in the supplemental material). We found that each isolate carried one *kan* gene in which the internal stop codon had been changed to its original sequence. Additionally, we screened all isolates by PCR for the occurrence of a chromosomal inversion involving the recombination cassettes. Previous studies on the inversely oriented *tuf* genes had shown that recombination between these inverted sequences could lead to a chromosomal inversion on the region between the homologous sequences in up to a quarter of recombinational events (18). In agreement with these data, we found that 8% (5 out of 59) of the selected kanamycin-resistant clones contained a chromosomal inversion between the recombination cassettes (Table S1). Taken together, these results show that the recombination cassettes can be used to measure the rate of recombinational repair between different locations on the bacterial chromosome. The limit of detection using these cassettes is in the range of 10^−8^ per cell per generation, as shown by the spontaneous kanamycin resistance rates.

**TABLE 1:** Rates of kanamycin resistance (recombination and mutation) as a function of genetic background

*cat*-*kan*(E3*) position (kb)	*amp*-*kan*(K138*) position (kb)	Strain^*a*^	Rate^*b*^	95% CI^*c*^
–450	+450	WT	4.5 × 10^−6^	3.4 × 10^−6^, 5.8 × 10^−6^
–450		WT	3.6 × 10^−9^	1.0 × 10^−9^, 6.9 × 10^−9^
	+450	WT	1.1 × 10^−9^	0.0, 2.6 × 10^−9^
–450	+450	Δ*recA* mutant	6.9 × 10^−9^	3.3 × 10^−9^, 1.3 × 10^−8^
–450	+450	Δ*recB* mutant	8.0 × 10^−8^	4.0 × 10^−8^, 1.5 × 10^−7^
–450	+450	Δ*recF* mutant	3.0 × 10^−6^	1.3 × 10^−6^, 5.5 × 10^−6^
–450	+450	Δ*recB* Δ*recF* mutant	4.3 × 10^−8^	1.5 × 10^−8^, 8.2 × 10^−8^

a Shown are mutations affecting recombination present in each strain. The wild type (WT) is recombination proficient.

b Rate of kanamycin resistance (combined effect of recombination plus mutation) per cell per generation.

c 95% confidence intervals (CI) for the rate of kanamycin resistance per cell per generation.

10.1128/mBio.01151-21.3 TABLE S1 Genotypes of 69 independent kanamycin-resistant isolates. Download Table S1, DOCX file, 0.03 MB. Copyright © 2021 Garmendia et al. 2021 Garmendia et al. https://creativecommons.org/licenses/by/4.0/ This content is distributed under the terms of the Creative Commons Attribution 4.0 International license.

### Recombinational repair is RecA and RecB dependent but RecF independent.

We next tested the potential underlying mechanisms of the observed recombinational repair (Fig. 1C). It was previously shown for the *tuf* genes that the rate of recombinational repair was strongly dependent on RecA and RecB function but not on RecF (7, 15). To test if recombinational repair rates between the *kan* cassettes were dependent on any of these three Rec protein functions, we constructed three strains that carried the two recombination cassettes and additionally a deletion of either *recA*, *recB*, or *recF* and measured the rates of recombinational repair (Table 1). As expected, deletion of *recA* reduced the rate of recombination repair 1,000-fold to a level that is indistinguishable from the spontaneous kanamycin resistance rates. Deletion of *recB* reduced recombination rates 100-fold but was still significantly higher than the spontaneous resistance rate, and the *recF* deletion did not lead to any decrease in recombination rate (Table 1). These data indicate that loss of RecA function fully abolishes recombination between the *kan* genes but that RecB function is responsible for only 90% of recombination events. It is conceivable that the remaining 10% of recombination events are RecF dependent. In this case, a RecF deletion would lead to only a small reduction in the recombination rate, which might not have been detected in the measurements. We therefore constructed a strain that had a combined *recB* and r*ecF* deletion and remeasured the recombinational repair rates. If the RecF function were to contribute to the recombinational repair rate, then we expected that the repair rate of the double deletion would be indistinguishable from the Δ*recA* rate and the spontaneous kanamycin resistance rates. Our results showed that the recombinational repair rate in a strain with a double deletion of *recB* and *recF* was indistinguishable from the rate in a *recB* single deletion strain, indicating that RecF function does not contribute to the recombination rate (Table 1). We isolated 10 kanamycin-resistant strains from the *recBF* double mutant and sequenced their *kan* genes to show that the kanamycin-resistant isolates represent genuine recombinants rather than external suppressor mutations. We found that each of the 10 isolates had a wild-type *kan* gene, as previously observed in recombination-proficient strains (Table S1). These results show that the recombinational repair of the *kan* genes is fully dependent on RecA function and that 90% of recombinational events follow the RecB pathway, whereas none of the recombination events follows the RecF pathway.

### Recombinational repair rates vary little across the chromosome.

**FIG 2:**
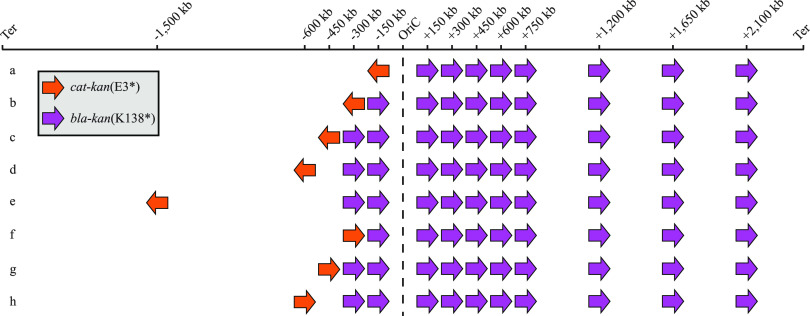
Overview of recombination cassette combinations. Eight sets of strains were constructed to measure recombinational repair rates between various parts of the bacterial chromosome. Each strain within a set has the *cat*-*kan*(E3*) cassette (orange) inserted in a fixed position of the chromosome and carries the *bla*-*kan*(K138*) cassette (purple) in one of 10 different positions. A total of 76 strains were constructed, and recombinational repair rates for each of the cassette combinations were measured. Ter, terminus.

After establishing that the constructed recombination cassettes could be used to measure recombinational repair rates between *kan* sequences located in different parts of the bacterial chromosome, we decided to include a larger number of chromosomal locations in the study. We left one of the *kan* cassettes at a fixed location (kb −450, on the left side of the chromosome) and moved the second cassette to a series of different locations in steps of 150 kb from that fixed location. After reaching the end of the first third of the right-hand side of the chromosome (kb + 750 from *oriC*), we increased the step size to 450 kb, placing additional insertions in the direction of the replication terminus (Fig. 2, set c). In total, 10 combinations of chromosomal locations were constructed (Fig. 2, set c), and recombinational repair rates between these positions were measured. The average recombination rate was 2.6 × 10^−6^ per cell per generation, and rates varied only 6-fold between the different positions (from 0.8 × 10^−6^ to 4.5 × 10^−6^ per cell per generation) (Table 2). Since these recombinational repair rates showed an unexpectedly low variation, we decided to further increase the number of chromosomal positions. This time, we moved the position of the recombination cassette that was previously fixed at kb −450 to four additional locations, three close to the origin of replication (kb −150, kb −300, and kb −600) and one about half way between the origin and terminus of replication (kb −1500). Each of the four new locations were combined with the 10 positions of the second recombination cassette. This increased the number of combinations of chromosomal locations to 47 (Fig. 2, sets a to e). We measured recombinational repair rates and found that the average recombination rate across all 47 combinations was unchanged at 2.6 × 10^−6^ per cell per generation. The variation increased to 45-fold between the lowest and highest rates (from 0.3 × 10^−6^ to 11.5 × 10^−6^ per cell per generation), but two outliers with rather high recombinational repair rates (9.5 × 10^−6^ and 11.5 × 10^−6^ per cell per generation) were responsible for the majority of the variation (Table 2). Excluding these two outlying values reduces the variation to 18-fold. The data for recombinational repair involving these 47 combinations of chromosomal positions show that any two locations of the chromosome that were tested could recombine with each other and that the rates of recombinational repair vary only by approximately 1 order of magnitude.

**TABLE 2:** Rates of recombinational repair^*a*^ between various locations^*b*^ on the chromosome

*cat*-*kan*(E3*) position (kb)	Rate of recombinational repair with *amp*-*kan*(K138*) at position (kb):									
	–300′	–150′	+150	+300	+450	+600	+750	+1200	+1650	+2100
−150			3.0	2.6	11.5	4.6	9.5	3.0	1.8	4.0
−300		4.0	2.6	1.8	4.6	4.6	3.5	1.8	3.0	4.0
−450	3.5	1.8	1.8	0.8	4.5	3.5	1.8	3.5	2.2	3.0
−600	1.1	1.1	0.5	0.3	1.8	2.6	0.5	0.7	2.6	0.8
−1500	1.4	0.5	1.1	0.8	3.5	1.4	0.8	1.4	1.4	1.8

a All values are shown as times 10^−6^ per cell per generation. See Table S2 for 95% confidence intervals.

b The insertion site of the *kan* cassette in the Salmonella chromosome in kilobases relative to *oriC*. Negative values correspond to locations on the left replichore, and positive values correspond to locations on the right replichore. All recombination cassettes are inserted in the direction of replication unless indicated otherwise by a prime symbol after the location number.

10.1128/mBio.01151-21.4 TABLE S2 Recombinational repair rates across the chromosome. Download Table S2, DOCX file, 0.04 MB. Copyright © 2021 Garmendia et al. 2021 Garmendia et al. https://creativecommons.org/licenses/by/4.0/ This content is distributed under the terms of the Creative Commons Attribution 4.0 International license.

### Each chromosomal position independently contributes to the recombinational repair rate.

**FIG 3:**
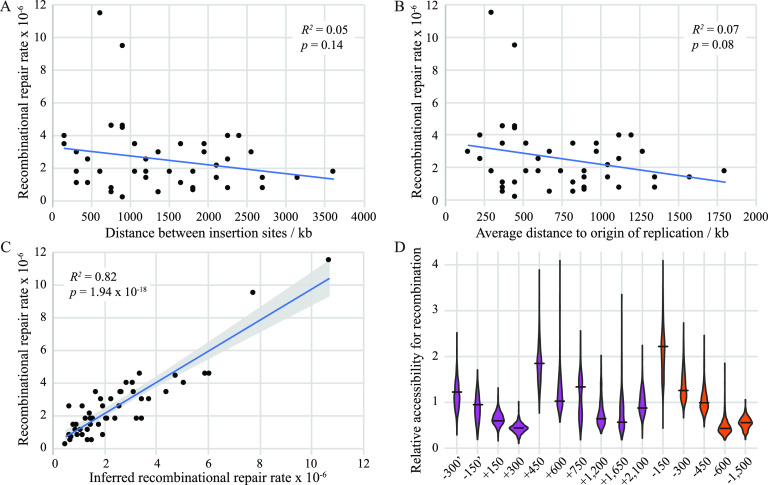
Analysis of recombinational repair rates across the chromosome. (A and B) Recombinational repair rates as a function of distance between the two insertion sites (A) and as an average distance between the insertion sites to the origin of replication (B). (C) Correlation between the measured recombinational repair rates and the rates inferred from estimated individual accessibility values. The blue lines represent the linear regression analyses. The gray area in panel C represents a 95% confidence interval for the regression. (D) Robustness of the estimated accessibility values of each of the 15 chromosomal locations. The optimal value inferred by the optimization function is shown as a black line. The distribution of values obtained from 1,000 jackknife samples containing only half the data are shown as violin plots. Locations with the *bla*-*kan*(K138*) cassette are shown in purple, and locations with the *cat*-*kan*(E3*) cassette are shown in orange.

The recombinational repair rates of the 47 pairs of chromosomal locations vary little but nevertheless show that not all chromosomal locations recombine at the same rate. This is especially true for the two pairs that display a significantly higher recombination rate (Table 2). We asked which factors might explain the observed variation. First, we tested if the physical distance on the chromosome between the recombination cassettes influenced the rate of recombinational repair, but we found no correlation (*R^2^* = 0.05, *P = *0.14) (Fig. 3A). Next, we asked whether the distance of the cassettes to the origin of replication affects the recombinational repair rate. During active replication, segments of the chromosome that are close to the origin of replication should be present within the cell in more copies than segments closer to the terminus (19, 20). It is therefore possible that the higher effective gene copy number increases the chances of recombination, but our data showed no correlation between the average distance of the paired cassettes from the origin of replication and their recombinational repair rates (*R^2^* = 0.07, *P = *0.08) (Fig. 3B). Another possibility is that recombination cassettes that are diametrically opposite each other on each replichore display higher recombination rates. This might be explained by the fact that these positions should be temporally in the same state of replication, but no such correlation was observed in our data (*R^2^* = 0.01, *P = *0.44) (Fig. S1A).

10.1128/mBio.01151-21.1 FIG S1 (A) Recombinational repair rates as a function of deviation from juxtaposed positioning of the recombination cassettes on opposite sides of the origin of replication. (B) Correlation between relative accessibility for recombination estimated using the original 15 chromosomal locations (*n* = 15) and including the novel kb +1500 location (*n* = 16). The red dot represents the kb +1500 location that was calculated using the estimated accessibility values for the four *cat*-*kan*(E3*) cassettes in the series with 15 chromosomal locations. The blue lines represent the linear regression analyses. Download FIG S1, DOCX file, 0.4 MB. Copyright © 2021 Garmendia et al. 2021 Garmendia et al. https://creativecommons.org/licenses/by/4.0/ This content is distributed under the terms of the Creative Commons Attribution 4.0 International license.

We decided to test if each chromosomal location individually impacts the total recombination rate independently from the partner cassette. It was previously shown, using phage lambda integrase-mediated recombination as a probe, that different chromosomal locations in Salmonella are not equally accessible (21). To test this for homologous recombination, we assumed that the average recombinational repair rate of 2.6 × 10^−6^ per cell per generation represents the average rate of recombination between any two positions on the chromosome. We further assumed that each chromosomal location has a relative accessibility for recombination that is independent of the location of the partner cassette. If a cassette is located in a position that has a more than average accessibility (>1), it will increase the recombinational repair rate, while a location with lower than average accessibility (<1) will lead to a lower rate. The measured recombinational repair rate would then be the product of the recombinational accessibilities of the two positions and the average recombinational repair rate. We then tested whether we could determine relative accessibility values for each of the tested insertion sites using an optimization function and our measured recombinational repair rates (see Materials and Methods). We found that the recombinational repair rates inferred by the optimization function correlate well with the observed ones (*R*^2^ = 0.817; *P = *1.94 × 10^−18^) (Fig. 3C). The values for the individual contributions range from 0.43 to 2.22, varying by a 5-fold factor between the lowest and highest values (Table S3). To further confirm these estimated accessibility values, we (i) tested the robustness of the estimates using a jackknife approach and (ii) tested their power to determine accessibility values of chromosomal locations that were not part of the initial data set. For the jackknife approach, we estimated the 15 individual contributions from half the available data which were randomly sampled from the original data set 1,000 times (ensuring that one value for each location was present at least once). The jackknife method showed that the accessibility values can be robustly estimated even from only half of the data (Fig. 3D and Fig. S2). Next, we inserted a *bla*-*kan*(K138*) cassette into a novel chromosomal location (kb +1500 relative to the origin of replication), combined it with the four existing *cat*-*kan*(E3*) cassettes (kb −300, kb −450, kb −600, and kb −1500 from the origin of replication), and measured recombinational repair rates (Table S2) (the asterisk indicates that the codon is mutated to a nonsense codon—TAA in each case). Using the four new measured recombinational repair rates, the previously determined average recombination rate across the chromosome, and the calculated accessibility values for the four *cat*-*kan*(E3*) cassettes, we calculated the accessibility value for the novel kb +1500 location to be 0.62 ± 0.29. We then recalculated the relative accessibility values for each of the chromosomal locations using the optimization function and the combined measured recombinational repair rates (the 47 previously measured ones and the 4 new ones). The results showed that (i) the accessibility value for the novel kb +1500 location was estimated to be 0.62, which is identical to the value calculated above, and (ii) the new accessibility values for the all locations correlate very well (*R^2^* = 0.81, *P = *2.01 × 10^−6^) with the values determined without the novel kb +1500 location (Fig. S1B). Taken together, these results indicate that the chromosomal location of each recombination cassette influences the rate of recombination between the two cassettes independently of the location of the partner cassette.

10.1128/mBio.01151-21.2 FIG S2 Estimated accessibility values of each of the 15 chromosomal locations. The optimal value inferred by the optimization function is shown as a black line. The distribution of values obtained from 1,000 jackknife samples containing only half the data are shown as violin plots. Locations with the *bla*-*kan*(K138*) cassette are shown in purple, and locations with the *cat*-*kan*(E3*) cassette are shown in orange. The violin plots for locations kb −150 and kb −300 are displayed offset. Download FIG S2, DOCX file, 0.7 MB. Copyright © 2021 Garmendia et al. 2021 Garmendia et al. https://creativecommons.org/licenses/by/4.0/ This content is distributed under the terms of the Creative Commons Attribution 4.0 International license.

10.1128/mBio.01151-21.5 TABLE S3 Relative accessibility for recombination of the chromosomal insertion sites. Download Table S3, DOCX file, 0.03 MB. Copyright © 2021 Garmendia et al. 2021 Garmendia et al. https://creativecommons.org/licenses/by/4.0/ This content is distributed under the terms of the Creative Commons Attribution 4.0 International license.

### The nucleoid structure of the chromosome affects accessibility values.

**FIG 4:**
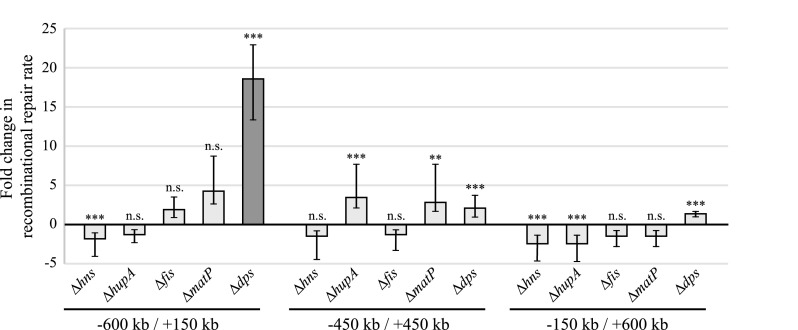
Fold changes in recombinational repair rates for strains carrying various NAP deletions. Positive values represent increased and negative values represent decreased recombinational repair rates. All values are relative to that for the isogenic strain without NAP deletion. The only strain that displayed a >5-fold change in recombinational repair rate is highlighted in dark gray. Locations of the recombination cassettes are indicated below. Significance was calculated using a chi-square test (n.s., nonsignificant; **, *P < *0.01; ***, *P < *0.001).

Our data suggest that regions within the Salmonella chromosome are not equally accessible for recombination. A possible explanation for this observation is that the three-dimensional structure of the chromosome affects recombination. Nucleoid-associated proteins (NAPs) bind to the DNA and aid chromosome condensation (22). In the chromosome’s condensed form, specific locations might be more or less exposed, which might affect recombination rates involving these locations. To test this hypothesis, we deleted different NAPs and tested if the lack of specific NAPs affected recombinational repair rates. We selected three strains that carry the recombination cassettes in six different chromosomal locations: (i) kb −600/kb +150, (ii) kb −450/kb +450, and (iii) kb −150/kb +600. Five NAP-encoding genes, *hns* (H-NS), *hupA* (HU), *fis* (Fis), *matP* (MatP), and *dps* (Dps), were inactivated for each of these three pairs of chromosomal locations, and the recombinational repair rates were measured for the resulting 15 strains (Fig. 4). We found that most NAP deletions had no significant effect (7 out of 15) or changed recombinational repair rates by only 2-fold to 3-fold (7 out of 15). Contrarily, the deletion of *dps* in the strain that carries the recombination cassettes in locations kb −600 and kb +150 led to an 18-fold increase in the recombinational repair rate. We compared the locations of the five insertion cassettes with known binding sites of Dps that were previously identified in Escherichia coli (23) and found that the only recombination cassette in close proximity to a potential Dps binding site is part of the pair that is affected by the *dps* deletion. The insertion site at kb −600 is only 181 bp apart from a potential Dps binding site, while all other insertion sites are at a 1.4- to 7.4-kb distance from potential Dps binding sites (Table S4). Strikingly, the insertion site at kb −600 was also determined to be the least accessible for recombination (Fig. 3D; Table S3). These data suggest that the nucleoid structure strongly affects the recombinational accessibility of the chromosomal location and is most likely responsible for the variation in accessibility among the various sites observed in this study.

10.1128/mBio.01151-21.6 TABLE S4 Distance between insertion sites and the closest potential Dps binding site. Download Table S4, DOCX file, 0.03 MB. Copyright © 2021 Garmendia et al. 2021 Garmendia et al. https://creativecommons.org/licenses/by/4.0/ This content is distributed under the terms of the Creative Commons Attribution 4.0 International license.

### Insertion of the recombination cassettes as direct repeats can increase the recombinational repair rates.

Next, we asked whether the relative sequence orientation of the *kan* cassettes affected recombination repair rates. All pairs of *kan* cassettes were originally constructed in the chromosome as inverted repeats (Fig. 2, sets a to e). We constructed a new set of strains with the *kan* genes as direct repeats (Fig. 2, sets f to h) to test whether this change would affect recombination rates. We chose three of the fixed *cat*-*kan*(E3*) cassette locations (kb −300, kb −450, and kb −600 from the origin of replication) and inserted the cassettes in the opposite direction. These cassettes were then combined with the 10 variable *amp*-*kan*(K138*) cassettes (Fig. 2, sets f to h). In total, 29 strains were constructed, and recombinational repair rates were measured as before. The average recombination rate was 3.9 × 10^−6^ per cell per generation and varied 12-fold between the various positions (from 0.8 × 10^−6^ per cell per generation to 9.5 × 10^−6^ per cell per generation) (Table S2). The average recombinational repair rate for the direct repeats is 2.2-fold higher than the average rate for the same set of inverted repeats (*P = *2.28 × 10^−4^, paired *t* test), which suggests that gene orientation has a significant impact on the recombinational repair rate.

## DISCUSSION

Bacterial chromosomes are composed largely of single-copy genes, but they frequently carry at least a few genes in multiple copies at separate chromosomal locations. In Salmonella, examples of such dispersed bacterial gene families that are both highly conserved and important for growth include the 7 *rrn* operons (rRNA), the duplicate *tuf* genes (EF-Tu), and genes for several different tRNA isoacceptor families (24). There is good evidence that genes within the *rrn* and *tuf* gene families coevolve by homologous recombination (7, 14, 16, 18, 25). However, because the functions of these genes, *rrn* and *tuf*, are highly important for growth and their chromosomal locations are also highly conserved, it is not obvious whether the rate of recombination between them is an evolved property particularly associated with these genes and their chromosomal locations.

Here, we tested the generality of intrachromosomal recombination by measuring rates of recombination between repetitive gene sequences placed at multiple different locations around the chromosome and in each orientation relative to the direction of replication. We asked whether every pair of sequences was capable of intrachromosomal recombination and whether the rates of recombination differed dramatically as a function of location or relative orientation. To address these questions, we constructed a genetic tool consisting of two mutationally inactivated kanamycin resistance genes that could be inserted into the chromosome at any desired location. The recombination cassettes were inserted into the chromosome of Salmonella in 81 different pairwise combinations to test different chromosomal locations and orientations, and recombinational repair rates were measured for each combination.

We could measure recombination between every gene pair constructed, with an average rate of 2.6 × 10^−6^ per cell per generation for gene pairs inserted in the inverted orientation and 3.9 × 10^−6^ per cell per generation for insertions in the direct orientation (see Table S2 in the supplemental material). The rate of recombination is approximately 1,000-fold above the spontaneous mutation rate of mutational reversion for nonsense mutations in each *kan* gene (Table 1). We also showed that recombination between the *kan* genes was completely dependent on RecA activity and largely (90%) dependent on RecB activity (Table 1), suggesting that the mechanism generating an active *kan* gene involves homologous recombination to repair chromosomal double-strand DNA (dsDNA) breaks. In this scenario, the break would have to occur within either of the *kan* genes, and processing of the dsDNA break by RecBCD must not proceed outside the recombination cassette; otherwise, the lack of homology would prevent homologous recombination. The presence of correctly oriented Chi sites may stop RecBCD-dependent processing within the recombination cassette and greatly facilitate recombinational repair (26, 27). The homologous regions within the two recombination cassettes used in this study are devoid of Chi or Chi-like sites, so recombinational repair must follow a Chi-independent pathway (14, 28, 29).

Recombination repair rates were, on average, 2.2-fold higher for *kan* genes in the direct orientation than for the same genes in the inverse orientation (Table S2). A possible explanation for this observation is that the gene orientation affects the probability of double-stranded DNA breaks to occur within the *kan* genes, which is required to stimulate homologous recombination. When the *kan* genes are inserted as inverted repeats, the *cat*-*kan*(E3*) cassette is oriented in the same direction as DNA replication (Fig. 2, sets a to e), while insertion as direct repeats turns the *cat*-*kan*(E3*) cassette in the opposite direction of DNA replication (Fig. 2, sets f to h). It is possible that clashes of RNA polymerases that transcribe the *kan* gene with the DNA polymerase that replicates the chromosome lead to an increased formation of double-stranded DNA breaks within the *kan* genes (30, 31).

SMC (structural maintenance of chromosomes) condensin complexes play a critical role in chromosome dynamics and have recently been shown in Bacillus subtilis to load at sites adjacent to the origin of replication and to tether the left and right arms of the chromosome together while travelling from the origin to the terminus (32). Because this process aligns left and right chromosome arms, it may increase the potential for recombination between sequences that are juxtaposed on opposite sides of the origin of replication. We asked whether potential juxtaposition of homologous sequences influenced the rate of recombination between *kan* sequences that were juxtaposed on opposite sides of the origin of replication and those that were misaligned with each other. Our data showed that there was no such correlation (Fig. S1A). We conclude that in Salmonella, potential alignment of left and right chromosome arms during replication has no significant effect on these recombinational repair rates.

Chromosomes are highly compacted to fit within bacterial cells and must be organized such that they can be efficiently replicated during each cell cycle and such that each part is accessible for transcription. Studies on the organization of bacterial chromosomes support the existence of multiple independent spatial domains composed of supercoiled plectonomes *in vivo* (33, 34). Nucleoid-associated proteins that bind DNA at specific sites and change their conformation are highly abundant and crucially important for chromosome condensation (22). Our data suggest that binding of NAPs to the DNA can have a profound impact on the recombinational accessibility of that chromosomal location. We found that binding of Dps significantly reduces the recombinational accessibility of one of the tested chromosomal locations (kb −600) in close proximity to the Dps binding site. Deletion of other NAPs also had a minor impact on recombination, leading to both an increased and a decreased repair rate (Fig. 4). These data suggest that the local three-dimensional structure of the DNA is a major determining factor for successful recombination. Our results also agree with a newly suggested model for a RecA-mediated homology search. In this model, a RecA-single-stranded DNA (ssDNA) filament simultaneously probes multiple different short dsDNA stretches to identify a homologous site, thus avoiding dependence on specific sites that are less available for recombination (35). In our experimental setup, homology for recombination does not extend beyond the kanamycin resistance genes, resulting in a dependence on the local chromosome structure for efficient recombination. Our data suggest that the bacterial chromosome is not homogenous with regard to homologous recombination but that the three-dimensional structure of the nucleoid creates regions that are more or less accessible for recombination.

## MATERIALS AND METHODS

### Bacterial strains and strain constructions.

All strains are derivatives of Salmonella enterica serovar Typhimurium strain LT2 (24). The *cat*-*kan*/*amp*-*kan* recombination cassettes and the *tetRA* genes for gene deletions were inserted into the Salmonella chromosome using dsDNA recombineering (36). All recombineering primers are shown in Tables S5 and S6 in the supplemental material. Genetic markers were moved between strains by P22 HT phage-mediated transduction (37).

10.1128/mBio.01151-21.7 TABLE S5 Oligonucleotides used to insert recombination cassettes into the chromosome. Download Table S5, DOCX file, 0.03 MB. Copyright © 2021 Garmendia et al. 2021 Garmendia et al. https://creativecommons.org/licenses/by/4.0/ This content is distributed under the terms of the Creative Commons Attribution 4.0 International license.

10.1128/mBio.01151-21.8 TABLE S6 Oligonucleotides used to delete various genes on the chromosome. Download Table S6, DOCX file, 0.03 MB. Copyright © 2021 Garmendia et al. 2021 Garmendia et al. https://creativecommons.org/licenses/by/4.0/ This content is distributed under the terms of the Creative Commons Attribution 4.0 International license.

### Bacterial growth conditions.

Bacteria were grown in LB medium (10% tryptone, 5% yeast extract [Oxoid, Basingstoke, England] and 10% NaCl [Merck, Darmstadt, Germany]) or on LA plates (LB with 1.5% agar [Oxoid]). Where indicated, the medium was supplemented with kanamycin (50 mg/liter), ampicillin (100 mg/liter), or chloramphenicol (25 mg/liter) (Sigma-Aldrich).

### Measuring recombination rates.

Independent cultures were inoculated in 15-ml tubes containing 2 ml LB, which were grown with shaking at 37°C to a final cell density of approximately 2 × 10^9^ cells ml^−1^. An appropriate number of cells (Table S7) was plated on LA plates with kanamycin and incubated overnight at 37°C. Recombination rates were calculated according to the formula μ = –(1/*n*)ln(P_0_), where μ is the recombination rate, *n* is the number of viable cells plated, and P_0_ is the proportion of cultures where no recombinants were scored. The initial number of independent cultures for each strain was 20. An additional 20 independent cultures were plated when the initial cultures did not contain at least two positive and two negative cultures.

10.1128/mBio.01151-21.9 TABLE S7 Number of cells plated in each fluctuation assay. Download Table S7, DOCX file, 0.03 MB. Copyright © 2021 Garmendia et al. 2021 Garmendia et al. https://creativecommons.org/licenses/by/4.0/ This content is distributed under the terms of the Creative Commons Attribution 4.0 International license.

### PCR and DNA sequencing.

PCRs for recombineering were done using Phusion high-fidelity PCR master mix (New England Biolabs, Ipswich, MA, USA). When screening for inversions and when preparing DNA for sequencing, we used the *Taq* polymerase-based PCR master mix from Thermo Scientific (Waltham, USA). Reactions were run on a model S1000 thermal cycler from Bio-Rad (Hercules, CA, USA). Oligonucleotides for PCR were purchased from Sigma-Aldrich. The denaturation temperature for PCRs was 98°C or 95°C for Phusion or *Taq* polymerase, respectively. The primer annealing temperature was 5°C below the calculated primer melting temperature, and the elongation time was set as 1 min per kb of product length. PCR products were prepared using the QIAquick PCR purification kit (Qiagen). Local sequencing was carried out by Macrogen Incorporated, Amsterdam, The Netherlands. The software CLC Main Workbench (7.7.2) from Qiagen was used for primer design, sequence analysis, and sequence comparisons.

### Calculation of relative accessibility values.

A model was built in R 4.0.2 (38) in which each of the 47 observed recombinational repair rates could be calculated as the product of the individual accessibility values of the two regions involved according to the formula μ*_a_*_,_*_b_* = *A_a_* × *A_b_* × μ_chr_, where μ*_a_*_,_*_b_* is the recombinational repair rate between locations *a* and *b*, *A_a_* and *A_b_* are the relative accessibilities for the recombination of chromosomal locations *a* and *b*, and μ_chr_ is the average recombination repair rate of all tested combinations of chromosomal locations. A quasi-Newton method allowing box constraints (39) (L-BFGS-B in the optim function of the stats package, with lower constraints set to 0 for all 15 variables) was used to minimize the sum of squares of the differences between the observed recombinational repair rates and the rates calculated from the products of the relative accessibility values.

To estimate the robustness of the estimates, we used a jackknife approach, estimating the 15 relative accessibility values from half the available data. We randomly sampled the original data set 1,000 times, ensuring that one value for each individual accessibility value was present at least once in each sample. The same optimization function was used on the sampled data to obtain estimates of individual regional contributions. The results were plotted using the R package ggplot2 3.3.2 (40).

### Statistical analysis.

Regression analysis to test correlations was performed using R, version 3.5.0 (38).
