# Supplementary material for: Chromosomal Location Determines the Rate of Intrachromosomal Homologous Recombination in Salmonella
Source: mBio. 2021 Jun 1;12(3):e01151-21. doi: 10.1128/mBio.01151-21 (PMC8262849; doi:10.1128/mBio.01151-21)
Supplement: TABLE S1 [file mbio.01151-21-st001.docx]

**TABLE S1** Genotypes of 69 independent kanamycin-resistant isolates.

| ***cat*-*kan*(E3*)**^a^ | | ***amp*-*kan*(K138*)**^a^ | | **Inversion**^b^ | **N_wt_**^c^ | **N_∆recBF_**^c^ |
| --- | --- | --- | --- | --- | --- | --- |
| **E3** | **K138** | **E3** | **K138** |  |  |  |
| wt | wt | wt | stop | No | 21 | 6 |
| stop | wt | wt | wt | No | 33 | 4 |
| stop | wt | wt | wt | Yes | 2 | 0 |
| stop | stop | wt | wt | Yes | 3 | 0 |

^a^ These recombination cassettes, each carrying an internal stop codon (E3* or K138*) were inserted on the leading strand of the chromosome for replication, on opposite sides of the replication origin, with each 450 kb from *oriC* (Fig. 1B). Stop indicates that the relevant stop codon was present in the kanamycin-resistant clone, whereas wt indicates that the relevant stop codon had been replaced by the wild-type codon thus generating an active enzyme and accounting for the kanamycin resistance phenotype.

^b^ Inversion was confirmed by PCR.

^c^ Number of independent kanamycin resistant isolates with the described genotype recovered in a recombination-proficient (wt) or ∆*recBF* background.
