# Supplementary material for: Chromosomal Location Determines the Rate of Intrachromosomal Homologous Recombination in Salmonella
Source: mBio. 2021 Jun 1;12(3):e01151-21. doi: 10.1128/mBio.01151-21 (PMC8262849; doi:10.1128/mBio.01151-21)
Supplement: TABLE S2 [file mbio.01151-21-st002.docx]

**TABLE S2** Recombinational repair rates across the chromosome^a^.

| **Strain** | ***cat*-*kan*(E3*)** | ***amp*-*kan*(K148*)** | **Rec^b^** | **Rate^c^** | **95% CI^d^** |
| --- | --- | --- | --- | --- | --- |
| TH10605 | -450 | +450 | wt | 4.5 x 10^-6^ | [3.4 x 10^-6^, 5.8 x 10^-6^] |
| TH10567 | -450 | - | wt | 3.6 x 10^-9^ | [1.0 x 10^-9^, 6.9 x 10^-9^] |
| TH10570 | - | +450 | wt | 1.1 x 10^-9^ | [0, 2.6 x 10^-9^] |
| TH10788 | -450 | +450 | ∆*recA* | 6.9 x 10^-9^ | [3.3 x 10^-9^, 1.3 x 10^-8^] |
| TH10789 | -450 | +450 | ∆*recB* | 8.0 x 10^-8^ | [4.0 x 10^-8^, 1.5 x 10^-7^] |
| TH10790 | -450 | +450 | ∆*recF* | 3.0 x 10^-6^ | [1.3 x 10^-6^, 5.5 x 10^-6^] |
| TH10853 | -450 | +450 | ∆*recBF* | 4.3 x 10^-8^ | [1.5 x 10^-8^, 8.2 x 10^-8^] |
| TH10740 | -150 | +450 | wt | 3.0 x 10^-6^ | [1.3 x 10^-6^, 5.5 x 10^-6^] |
| TH10741 | -150 | +450 | wt | 2.6 x 10^-6^ | [1.0 x 10^-6^, 4.8 x 10^-6^] |
| TH10742 | -150 | +450 | wt | 1.2 x 10^-5^ | [7.3 x 10^-6^, >1.5 x 10^-5^] |
| TH10743 | -150 | +600 | wt | 4.6 x 10^-6^ | [2.4 x 10^-6^, 8.4 x 10^-6^] |
| TH10811 | -150 | +750 | wt | 9.5 x 10^-6^ | [5.9 x 10^-6^, >1.5 x 10^-5^] |
| TH10744 | -150 | +1,200 | wt | 3.0 x 10^-6^ | [1.3 x 10^-6^, 5.5 x 10^-6^] |
| TH10747 | -150 | +1,500 | wt | 1.4 x 10^-6^ | [3.1 x 10^-7^, 2.9 x 10^-6^] |
| TH10745 | -150 | +1,650 | wt | 1.8 x 10^-6^ | [5.2 x 10^-7^, 3.5 x 10^-6^] |
| TH10746 | -150 | +2,100 | wt | 4.0 x 10^-6^ | [2.0 x 10^-6^, 7.3 x 10^-6^] |
| TH10762 | -300 | -150' | wt | 4.0 x 10^-6^ | [2.0 x 10^-6^, 7.3 x 10^-6^] |
| TH10692 | -300 | +150 | wt | 2.6 x 10^-6^ | [1.0 x 10^-6^, 4.8 x 10^-6^] |
| TH10693 | -300 | +300 | wt | 1.8 x 10^-6^ | [5.2 x 10^-7^, 3.5 x 10^-6^] |
| TH10694 | -300 | +450 | wt | 4.6 x 10^-6^ | [2.4 x 10^-6^, 8.4 x 10^-6^] |
| TH10695 | -300 | +600 | wt | 4.6 x 10^-6^ | [2.4 x 10^-6^, 8.4 x 10^-6^] |
| TH10812 | -300 | +750 | wt | 3.5 x 10^-6^ | [1.6 x 10^-6^, 6.3 x 10^-6^] |
| TH10696 | -300 | +1,200 | wt | 1.8 x 10^-6^ | [5.2 x 10^-7^, 3.5 x 10^-6^] |
| TH10699 | -300 | +1,500 | wt | 1.4 x 10^-6^ | [3.1 x 10^-7^, 2.9 x 10^-6^] |
| TH10697 | -300 | +1,650 | wt | 3.0 x 10^-6^ | [1.3 x 10^-6^, 5.5 x 10^-6^] |
| TH10698 | -300 | +2,100 | wt | 4.0 x 10^-6^ | [2.0 x 10^-6^, 7.3 x 10^-6^] |
| TH10899 | -300' | -150' | wt | 5.2 x 10^-6^ | [2.9 x 10^-6^, 9.8 x 10^-6^] |
| TH10900 | -300' | +150 | wt | 8.0 x 10^-6^ | [4.9 x 10^-6^, 1.9 x 10^-5^] |
| TH10901 | -300' | +300 | wt | 2.6 x 10^-6^ | [1.0 x 10^-6^, 4.8 x 10^-6^] |
| TH10902 | -300' | +450 | wt | 8.0 x 10^-6^ | [4.9 x 10^-6^, 1.9 x 10^-5^] |
| TH10903 | -300' | +600 | wt | 6.0 x 10^-6^ | [3.5 x 10^-6^, 1.2 x 10^-5^] |
| TH10904 | -300' | +750 | wt | 9.5 x 10^-6^ | [5.9 x 10^-6^, >1.5 x 10^-5^] |
| TH10905 | -300' | +1,200 | wt | 3.5 x 10^-6^ | [1.6 x 10^-6^, 6.3 x 10^-6^] |
| TH10907 | -300' | +1,650 | wt | 1.4 x 10^-6^ | [3.1 x 10^-7^, 2.9 x 10^-6^] |
| TH10908 | -300' | +2,100 | wt | 4.6 x 10^-6^ | [2.4 x 10^-6^, 8.4 x 10^-6^] |
| TH10756 | -450 | -300' | wt | 3.5 x 10^-6^ | [1.6 x 10^-6^, 6.3 x 10^-6^] |
| TH10920 | -450 | -150' | wt | 1.8 x 10^-6^ | [5.2 x 10^-7^, 3.5 x 10^-6^] |
| TH10603 | -450 | +150 | wt | 1.8 x 10^-6^ | [5.2 x 10^-7^, 3.5 x 10^-6^] |
| TH10604 | -450 | +300 | wt | 8.1 x 10^-7^ | [0, 1.8 x 10^-6^] |
| TH10606 | -450 | +600 | wt | 3.5 x 10^-6^ | [1.6 x 10^-6^, 6.3 x 10^-6^] |
| TH10813 | -450 | +750 | wt | 1.8 x 10^-6^ | [5.2 x 10^-7^, 3.5 x 10^-6^] |
| TH10607 | -450 | +1,200 | wt | 3.5 x 10^-6^ | [1.6 x 10^-6^, 6.3 x 10^-6^] |
| TH10645 | -450 | +1,500 | wt | 1.8 x 10^-6^ | [5.2 x 10^-7^, 3.5 x 10^-6^] |
| TH10608 | -450 | +1,650 | wt | 2.2 x 10^-6^ | [7.6 x 10^-7^, 4.1 x 10^-6^] |
| TH10609 | -450 | +2,100 | wt | 3.0 x 10^-6^ | [1.3 x 10^-6^, 5.5 x 10^-6^] |
| TH10757 | -450' | -300' | wt | 8.0 x 10^-6^ | [4.9 x 10^-6^, 1.9 x 10^-5^] |
| TH10764 | -450' | -150' | wt | 8.1 x 10^-7^ | [0, 1.8 x 10^-6^] |
|  |  |  |  |  |  |
| **TABLE S2** Continued. | | | | | |
| TH10748 | -450' | +150 | wt | 6.0 x 10^-6^ | [3.5 x 10^-6^, 1.2 x 10^-5^] |
| TH10749 | -450' | +300 | wt | 2.2 x 10^-6^ | [7.6 x 10^-7^, 4.1 x 10^-6^] |
| TH10750 | -450' | +450 | wt | 4.6 x 10^-6^ | [2.4 x 10^-6^, 8.4 x 10^-6^] |
| TH10751 | -450' | +600 | wt | 3.5 x 10^-6^ | [1.6 x 10^-6^, 6.3 x 10^-6^] |
| TH10814 | -450' | +750 | wt | 1.8 x 10^-6^ | [5.2 x 10^-7^, 3.5 x 10^-6^] |
| TH10752 | -450' | +1,200 | wt | 4.0 x 10^-6^ | [2.0 x 10^-6^, 7.3 x 10^-6^] |
| TH10753 | -450' | +1,650 | wt | 2.2 x 10^-6^ | [7.6 x 10^-7^, 4.1 x 10^-6^] |
| TH10754 | -450' | +2,100 | wt | 2.6 x 10^-6^ | [1.0 x 10^-6^, 4.8 x 10^-6^] |
| TH10758 | -600 | -300' | wt | 1.1 x 10^-6^ | [1.3 x 10^-7^, 2.4 x 10^-6^] |
| TH10765 | -600 | -150' | wt | 1.1 x 10^-6^ | [1.3 x 10^-7^, 2.4 x 10^-6^] |
| TH10711 | -600 | +150 | wt | 5.3 x 10^-7^ | [0, 1.3 x 10^-6^] |
| TH10712 | -600 | +300 | wt | 2.6 x 10^-7^ | [0, 6.3 x 10^-7^] |
| TH10713 | -600 | +450 | wt | 1.8 x 10^-6^ | [5.2 x 10^-7^, 3.5 x 10^-6^] |
| TH10714 | -600 | +600 | wt | 2.6 x 10^-6^ | [1.0 x 10^-6^, 4.8 x 10^-6^] |
| TH10815 | -600 | +750 | wt | 5.3 x 10^-7^ | [0, 1.3 x 10^-6^] |
| TH10715 | -600 | +1,200 | wt | 6.7 x 10^-7^ | [1.1 x 10^-7^, 1.3 x 10^-6^] |
| TH10718 | -600 | +1,500 | wt | 8.1 x 10^-7^ | [0, 1.8 x 10^-6^] |
| TH10716 | -600 | +1,650 | wt | 2.6 x 10^-6^ | [1.0 x 10^-6^, 4.8 x 10^-6^] |
| TH10717 | -600 | +2,100 | wt | 8.1 x 10^-7^ | [0, 1.8 x 10^-6^] |
| TH10909 | -600' | -300' | wt | 4.6 x 10^-6^ | [2.4 x 10^-6^, 8.4 x 10^-6^] |
| TH10910 | -600' | -150' | wt | 5.2 x 10^-6^ | [2.9 x 10^-6^, 9.8 x 10^-6^] |
| TH10911 | -600' | +150 | wt | 3.5 x 10^-6^ | [1.6 x 10^-6^, 6.3 x 10^-6^] |
| TH10912 | -600' | +300 | wt | 1.1 x 10^-6^ | [1.3 x 10^-7^, 2.4 x 10^-6^] |
| TH10913 | -600' | +450 | wt | 2.2 x 10^-6^ | [7.6 x 10^-7^, 4.1 x 10^-6^] |
| TH10914 | -600' | +600 | wt | 2.2 x 10^-6^ | [7.6 x 10^-7^, 4.1 x 10^-6^] |
| TH10915 | -600' | +750 | wt | 2.2 x 10^-6^ | [7.6 x 10^-7^, 4.1 x 10^-6^] |
| TH10916 | -600' | +1,200 | wt | 1.4 x 10^-6^ | [3.1 x 10^-7^, 2.9 x 10^-6^] |
| TH10918 | -600' | +1,650 | wt | 1.1 x 10^-6^ | [1.3 x 10^-7^, 2.4 x 10^-6^] |
| TH10919 | -600' | +2,100 | wt | 1.1 x 10^-6^ | [1.3 x 10^-7^, 2.4 x 10^-6^] |
| TH10759 | -1,500 | -300' | wt | 1.4 x 10^-6^ | [3.1 x 10^-7^, 2.9 x 10^-6^] |
| TH10766 | -1,500 | -150' | wt | 5.3 x 10^-7^ | [0, 1.3 x 10^-6^] |
| TH10700 | -1,500 | +150 | wt | 1.1 x 10^-6^ | [1.3 x 10^-7^, 2.4 x 10^-6^] |
| TH10701 | -1,500 | +300 | wt | 8.1 x 10^-7^ | [0, 1.8 x 10^-6^] |
| TH10702 | -1,500 | +450 | wt | 3.5 x 10^-6^ | [1.6 x 10^-6^, 6.3 x 10^-6^] |
| TH10703 | -1,500 | +600 | wt | 1.4 x 10^-6^ | [3.1 x 10^-7^, 2.9 x 10^-6^] |
| TH10816 | -1,500 | +750 | wt | 8.1 x 10^-7^ | [0, 1.8 x 10^-6^] |
| TH10704 | -1,500 | +1,200 | wt | 1.4 x 10^-6^ | [3.1 x 10^-7^, 2.9 x 10^-6^] |
| TH10707 | -1,500 | +1,500 | wt | 1.4 x 10^-6^ | [3.1 x 10^-7^, 2.9 x 10^-6^] |
| TH10705 | -1,500 | +1,650 | wt | 1.4 x 10^-6^ | [3.1 x 10^-7^, 2.9 x 10^-6^] |
| TH10706 | -1,500 | +2,100 | wt | 1.8 x 10^-6^ | [5.2 x 10^-7^, 3.5 x 10^-6^] |

^a^ Insertion site in the *Salmonella* chromosome in kb relative to *oriC*. Negative values correspond to locations on the left replichore and positive values to the right replichore. All recombination cassettes are inserted in the direction of replication unless indicated otherwise by the notation ‘ after the location number.

^b^ Mutations affecting recombination present in each strain. wt is recombination proficient.

^c^ Rate of kanamycin resistance (combined effect of recombination plus mutation) per cell per generation.

^d^ 95% confidence intervals for the rate of kanamycin resistance per cell per generation.
