# Supplementary material for: Chromosomal Location Determines the Rate of Intrachromosomal Homologous Recombination in Salmonella
Source: mBio. 2021 Jun 1;12(3):e01151-21. doi: 10.1128/mBio.01151-21 (PMC8262849; doi:10.1128/mBio.01151-21)
Supplement: FIG S1 [file mbio.01151-21-sf001.docx]

**FIG S1** (**A**) Recombinational repair rates as a function of deviation from juxtaposed positioning of the recombination cassettes on opposite sides of the origin of replication. (**B**) Correlation between relative accessibility for recombination estimated using the original fifteen chromosomal locations (N = 15) and including the novel +1,500 kb location (N = 16). The red dot represents the +1,500 kb location that was calculated using the estimated accessibility values for the four *cat*-*kan*(E3*) cassettes in the N =15 series. The blue lines represent the linear regression analyses.
