# Supplementary material for: Chromosomal Location Determines the Rate of Intrachromosomal Homologous Recombination in Salmonella
Source: mBio. 2021 Jun 1;12(3):e01151-21. doi: 10.1128/mBio.01151-21 (PMC8262849; doi:10.1128/mBio.01151-21)
Supplement: FIG S2 [file mbio.01151-21-sf002.docx]

**FIG S2** Estimated accessibility values of each of the 15 chromosomal locations. The optimal value inferred by the optimization function is shown as a black line. The distribution of values obtained from 1,000 jackknife samples containing only half the data is shown as violin plots. Locations with the *bla*-*kan*(K138*) cassette are shown in purple and locations with the *cat*-*kan*(E3*) cassette are shown in orange. The violin plots for locations -150 kb and -300 kb are displayed off-set.
