# Supplementary material for: Chromosomal Location Determines the Rate of Intrachromosomal Homologous Recombination in Salmonella
Source: mBio. 2021 Jun 1;12(3):e01151-21. doi: 10.1128/mBio.01151-21 (PMC8262849; doi:10.1128/mBio.01151-21)
Supplement: TABLE S3 [file mbio.01151-21-st003.docx]

**TABLE S3** Relative accessibility for recombination of the chromosomal insertion sites^a^.

| ***amp*-*kan*(K138*)** | | | ***cat*-*kan*(E3*)** | | |
| --- | --- | --- | --- | --- | --- |
| **Location** | **Accessibility^b^** | | **Location** | **Accessibility^b^** | |
|  | **N=15** | **N=16** |  | **N=15** | **N=16** |
| -300’ | 1.23 | 1.63 | -150 | 2.22 | 1.87 |
| -150’ | 0.95 | 0.78 | -300 | 1.26 | 1.07 |
| +150 | 0.60 | 0.69 | -450 | 0.99 | 0.92 |
| +300 | 0.45 | 0.67 | -600 | 0.43 | 0.35 |
| +450 | 1.85 | 2.39 | -1,500 | 0.56 | 0.52 |
| +600 | 1.02 | 1.37 |  |  |  |
| +750 | 1.34 | 1.58 |  |  |  |
| +1,200 | 0.65 | 0.82 |  |  |  |
| +1,500 | 0.62 ± 0.29^c^ | 0.62 |  |  |  |
| +1,650 | 0.57 | 0.84 |  |  |  |
| +2,100 | 0.88 | 1.07 |  |  |  |

^a^ Insertion site in the *Salmonella* chromosome in kb relative to *oriC*. Negative values correspond to locations on the left replichore and positive values to the right replichore. All recombination cassettes are inserted in the direction of replication unless indicated otherwise by the notation ‘ after the location number.

^b^ Calculation of relative accessibility is described in Material and Methods. The N=15 dataset does not include the +1,500 kb location in the calculation.

^c^ The accessibility value was not calculated as part of the optimization function but using the measured recombinational repair rates and the accessibility values of the pairing *cat*-*kan*(E3*) cassettes.
