# Supplementary material for: Chromosomal Location Determines the Rate of Intrachromosomal Homologous Recombination in Salmonella
Source: mBio. 2021 Jun 1;12(3):e01151-21. doi: 10.1128/mBio.01151-21 (PMC8262849; doi:10.1128/mBio.01151-21)
Supplement: TABLE S4 [file mbio.01151-21-st004.docx]

**TABLE S4** Distance between insertion sites and closest potential Dps binding site.

| **Location^a^** | **Cassette** | **Distance to potential Dps binding site^b^** |
| --- | --- | --- |
| -600 kb | *cat*-*kan*(E3*) | 181 bp |
| -450 kb | *cat*-*kan*(E3*) | 2,118 bp |
| -150 kb | *cat*-*kan*(E3*) | 1,490 bp |
| +150 kb | *amp*-*kan*(K138*) | 7,446 bp |
| +450 kb | *amp*-*kan*(K138*) | 1,785 bp |
| +600 kb | *amp*-*kan*(K138*) | 2,247 bp |

^a^ Insertion site in the *Salmonella* chromosome in kb relative to *oriC*. Negative values correspond to locations on the left replichore and positive values to the right replichore. All recombination cassettes are inserted in the direction of replication.

^b^ Distance to closest Dps binding site identified in *E. coli*.
