# Supplementary material for: Chromosomal Location Determines the Rate of Intrachromosomal Homologous Recombination in Salmonella
Source: mBio. 2021 Jun 1;12(3):e01151-21. doi: 10.1128/mBio.01151-21 (PMC8262849; doi:10.1128/mBio.01151-21)
Supplement: TABLE S5 [file mbio.01151-21-st005.docx]

**TABLE S5** Oligonucleotides used to insert recombination cassettes into the chromosome.

| **Primer^a^** | **Sequence 5’>3’^b^** |
| --- | --- |
| -1,500f | TGGTCGACAATCTCATGGTCCGGCCGGTTGAACTGTGCGC/AAAATGAGACGTTGATCGGCACG |
| -1,500r | GCGAATGCCGTCGGGCTGGCGATTGCCGAGCGTACGTTAG/ATCAAAGGGAAAACTGTCCA |
| -600f | TTTCCAGCGCTTGCTGGAGTTCCTGCTGAAGTTCCAGCGT/AAAATGAGACGTTGATCGGCACG |
| -600r | GCCTCAGCTACAACAGGCCATCCGTCTGTTGCAGTTGTCT/ATCAAAGGGAAAACTGTCCA |
| -600’f | TTTCCAGCGCTTGCTGGAGTTCCTGCTGAAGTTCCAGCGT/ATCAAAGGGAAAACTGTCCA |
| -600’ | GCCTCAGCTACAACAGGCCATCCGTCTGTTGCAGTTGTCT/AAAATGAGACGTTGATCGGCACG |
| -450f | ATCCTGGCTACCCTGGGCGTTCCACACCACGCTGGTGGAA/AAAATGAGACGTTGATCGGCACG |
| -450r | CCTTCACCGGCAGCAATATCCAGGGCGCGGACAACATCAC/ATCAAAGGGAAAACTGTCCA |
| -450’f | ATCCTGGCTACCCTGGGCGTTCCACACCACGCTGGTGGAA/ATCAAAGGGAAAACTGTCCA |
| -450’r | CCTTCACCGGCAGCAATATCCAGGGCGCGGACAACATCAC/AAAATGAGACGTTGATCGGCACG |
| -300f | CATCAGCATCCACAGTATTTCATTGCGCGGCAAGCCAAGC/AAAATGAGACGTTGATCGGCACG |
| -300r | TCTATTCCATGACTTACAGCACGGCCGCCGCGCCGGTGGG/ATCAAAGGGAAAACTGTCCA |
| -300’f | CATCAGCATCCACAGTATTTCATTGCGCGGCAAGCCAAGC/ATCAAAGGGAAAACTGTCCA |
| -300’r | TCTATTCCATGACTTACAGCACGGCCGCCGCGCCGGTGGG/AAAATGAGACGTTGATCGGCACG |
| -150f | ATTATGGAAAAGTATGGCGTGGGGAATTTTATTGTGGCAT/AAAATGAGACGTTGATCGGCACG |
| -150r | TTGCTGGCGGCCAGGCCGGGCAAAGCGCCCCACTCTGCGA/ATCAAAGGGAAAACTGTCCA |
| -150’f | ATTATGGAAAAGTATGGCGTGGGGAATTTTATTGTGGCAT/ATCAAAGGGAAAACTGTCCA |
| -150’r | TTGCTGGCGGCCAGGCCGGGCAAAGCGCCCCACTCTGCGA/AAAATGAGACGTTGATCGGCACG |
| +150f | CGAAAATGCTTGAGGAATGACCATGGTTCGTATTGCTTGT/AAAATGAGACGTTGATCGGCACG |
| +150r | CTTCCACGTAATAGATGCGATCCATCACGGTAATACCTAC/ATCAAAGGGAAAACTGTCCA |
| +300f | TTGCGTCTGGAGCGGCTGGAAAGCTGGCAGCACGTTACCT/AAAATGAGACGTTGATCGGCACG |
| +300r | GCGTAGTTCGGGTACATGCGCTCGCACAAGCAGGCCATAA/ATCAAAGGGAAAACTGTCCA |
| +450f | TCAGAATGAGTACCGGCAGGGTATATTTTTTCTGTCGGAT/AAAATGAGACGTTGATCGGCACG |
| +450r | AGGGCTGCCCGATGAGGATGGCCTGCATTTCCTGACGCGA/ATCAAAGGGAAAACTGTCCA |
| +600f | TGTTTGATGAAATCTCAGCCGAATCAATGGCGCTCGCCCG/AAAATGAGACGTTGATCGGCACG |
| +600r | TTCTTCGTCAGGAAGGTTGCCAAACGCCGCCACGATTTCG/ATCAAAGGGAAAACTGTCCA |
| +750f | CGTGTTTTGCATTCTCTCCGGCAGCATGAACCAGAAATGA/AAAATGAGACGTTGATCGGCACG |
| +750r | CTTATCCGGCCTACGGTCGCCTTGCCATTGGGCAGGTTAA/ATCAAAGGGAAAACTGTCCA |
| +1,200f | TGATACCGAAGTATTCAGCACTGATAAACTGGAACTGACC/AAAATGAGACGTTGATCGGCACG |
| +1,200r | CAGAAACAACATTACCGTTGTGATCAGCAGTATTGCCGCT/ATCAAAGGGAAAACTGTCCA |
| +1,500f | CCGGTTAGCCACTTTACGGGCAAAGCCCATTTCGTGCGTC/AAAATGAGACGTTGATCGGCACG |
| +1,500r | TGGTCGAGCTGGCGAACGAAGGGATGACCATGATGGTGGT/ATCAAAGGGAAAACTGTCCA |
| +1,650f | ATGCTGAGTGAGAAAATGCGCGTGATAGTACTGCCGGGCA/AAAATGAGACGTTGATCGGCACG |
| +1,650r | GGGATCGGGATGCGTTACCGACTATACTCTCGCCTCGGGT/ATCAAAGGGAAAACTGTCCA |
| +2,100f | TAGAACTTTTAGCACTACAAGCAGACCAATTTATGCTGCA/AAAATGAGACGTTGATCGGCACG |
| +2,100r | AGGCGCTGGCACCTCCATTTTCACCGTTCAGAAAGTCCAG/ATCAAAGGGAAAACTGTCCA |

^a^ Insertion site in the *Salmonella* chromosome. Numbers indicate kb distance to the origin of replication. Negative values correspond to locations relative to *oriC* on the left replichore and positive values to the right replichore. All recombination cassettes are inserted in the direction of replication unless indicated otherwise by the notation ‘ after the location number.

^a^ The 5’ end of each primer contains 40 nt homology to the chromosomal insertion site. The 3’ end is homologous to the recombination cassettes.
