# Supplementary material for: Chromosomal Location Determines the Rate of Intrachromosomal Homologous Recombination in Salmonella
Source: mBio. 2021 Jun 1;12(3):e01151-21. doi: 10.1128/mBio.01151-21 (PMC8262849; doi:10.1128/mBio.01151-21)
Supplement: TABLE S6 [file mbio.01151-21-st006.docx]

**TABLE S6** Oligonucleotides used to delete various genes on the chromosome.

| **Primer** | **Sequence 5’>3’^a^** |
| --- | --- |
| ∆recAf | ATGGCGGCTTCGTTTTGCCCGCCCCACCATCACCTGATGAGAATTCGAGCTCGGTACCCG |
| ∆recAr | ACTATCCGGTTCAATACCAAGTTGCATGACAGGAGTAATAGCTATGACCATGATTACGCC |
| ∆recBf | CAAACGTTACCTCTAATGAGCGAAAAGAATGAATGATGTCGAATTCGAGCTCGGTACCCG |
| ∆recBr | ATTGTCATTGCGCCTCCTCACTCATTTCACCTGCAAACATGCTATGACCATGATTACGCC |
| ∆recFf | CCCTGGACGAATGTACTAATATATGTCACTGACGCGCCTTGAATTCGAGCTCGGTACCCG |
| ∆recFr | ACATGTCTATAACGTGTTCAGCGCTAATTGCGCTGACAAAGCTATGACCATGATTACGCC |
| ∆hnsf | ATGAGCGAAGCACTTAAAATTCTGAACAACATCCGTACTCTTCGTTATAGGAACTTCAGAGCG |
| ∆hnsr | TTATTCCTTGATCAGGAAATCTTCCAGTTGCTTACCTTGTTCTTCCAACAGCAATGGATCACT |
| ∆hupAf | ATGAACAAGACTCAACTGATTGATGTAATTGCAGACAAAGCAGAATATAGGAACTTCAGAGCG |
| ∆hupAr | TTACTTAACTGCGTCTTTCAGAGCTTTACCAGAAACAAACGCCGGCAACAGCAATGGATCACT |
| ∆fisf | ATGTTCGAACAACGCGTAAATTCTGACGTACTGACCGTTTCTACCTATAGGAACTTCAGAGCG |
| ∆fisr | TTAGTTCATGCCGTATTTTTTTAATTTTTTACGCAGCGTACCACGCAACAGCAATGGATCACT |
| ∆matPf | ATGAAATATCAACAACTTGAAAATCTTGAAAGCGGTTGGAAATGGTATAGGAACTTCAGAGCG |
| ∆matPr | TTACTTCTTGCCTAATAACGCCTGTAAATCCTGCTTTAACGTGGTCAACAGCAATGGATCACT |
| ∆dpsf | TTATTCGATGTTGGATTCGATAAACCACAGGAATTTATCGAGGTCTATAGGAACTTCAGAGCG |
| ∆dpsr | ATGAGTACCGCTAAACTGGTAAAAACAAAAGCGTCTAATCTGCTTCAACAGCAATGGATCACT |

^a^ The 5’ end of each primer contains 40 nt homology to the chromosomal insertion site. The 3’ end is homologous to the *tetRA* cassettes used for the gene deletion.
