# Supplementary material for: Chromosomal Location Determines the Rate of Intrachromosomal Homologous Recombination in Salmonella
Source: mBio. 2021 Jun 1;12(3):e01151-21. doi: 10.1128/mBio.01151-21 (PMC8262849; doi:10.1128/mBio.01151-21)
Supplement: TABLE S7 [file mbio.01151-21-st007.docx]

**TABLE S7** Number of cells plated in each fluctuation assay.

| **Strain background^a^** | **N (cfu)** |
| --- | --- |
| wt | 2 x 10^5^ |
| wt, single cassette | 1 x 10^8^ |
| ∆*recA* | 1 x 10^8^ |
| ∆*recB* | 1 x 10^7^ |
| ∆*recF* | 2 x 10^5^ |
| ∆*recB* + ∆*recF* | 1 x 10^7^ |

^a^ Mutations affecting recombination present in each strain (wt indicates recombination-proficient).
